# The Fine-Tuned Large Language Model for Extracting the Progressive Bone Metastasis from Unstructured Radiology Reports

**DOI:** 10.1007/s10278-024-01242-3

**Published:** 2024-08-26

**Authors:** Noriko Kanemaru, Koichiro Yasaka, Nana Fujita, Jun Kanzawa, Osamu Abe

**Affiliations:** Department of Radiology, Graduate School of Medicine, The University of Tokyo, 7-3-1 Hongo, Bunkyo-Ku, Tokyo, 113-8655 Japan

**Keywords:** Large language model, Bone metastasis, Deep learning

## Abstract

Early detection of patients with impending bone metastasis is crucial for prognosis improvement. This study aimed to investigate the feasibility of a fine-tuned, locally run large language model (LLM) in extracting patients with bone metastasis in unstructured Japanese radiology report and to compare its performance with manual annotation. This retrospective study included patients with “metastasis” in radiological reports (April 2018–January 2019, August–May 2022, and April–December 2023 for training, validation, and test datasets of 9559, 1498, and 7399 patients, respectively). Radiologists reviewed the clinical indication and diagnosis sections of the radiological report (used as input data) and classified them into groups 0 (no bone metastasis), 1 (progressive bone metastasis), and 2 (stable or decreased bone metastasis). The data for group 0 was under-sampled in training and test datasets due to group imbalance. The best-performing model from the validation set was subsequently tested using the testing dataset. Two additional radiologists (readers 1 and 2) were involved in classifying radiological reports within the test dataset for testing purposes. The fine-tuned LLM, reader 1, and reader 2 demonstrated an accuracy of 0.979, 0.996, and 0.993, sensitivity for groups 0/1/2 of 0.988/0.947/0.943, 1.000/1.000/0.966, and 1.000/0.982/0.954, and time required for classification (s) of 105, 2312, and 3094 in under-sampled test dataset (*n* = 711), respectively. Fine-tuned LLM extracted patients with bone metastasis, demonstrating satisfactory performance that was comparable to or slightly lower than manual annotation by radiologists in a noticeably shorter time.

## Background

The bone is one of the most predominantly involved sites of cancer metastasis after the lungs and liver [1]. Bone metastasis causes skeletal-related events (SREs), such as spinal cord compression and pathological bone fractures, that significantly deteriorate the activities of daily living, quality of life, and survival. Early diagnosis and succeeding prophylactic or therapeutic measures by multidisciplinary approach are crucial [1]. As physicians sometimes overlook signs of serious SREs such as pain, motor dysfunction, and sensory disturbance, radiological examinations and their reports play an important role in promptly detecting patients at risk of SREs [2]. However, there are two main problems. First, in some cases, radiology examinations or reports are not thoroughly reviewed in some cases [3, 4], delaying the detection of the impending status of patients. This highlights the importance of an alert system for radiological reports. Additionally, even when referring physicians recognize bone metastasis, they may not fully grasp the urgency of the clinical situation, resulting in delayed intervention [5, 6]. Kimura et al. emphasized the need for radiologists to identify the potential patient for serious SREs from all radiological studies of patients with advanced cancer and bring such cases to multidisciplinary discussions [2]. However, extracting such radiological exams from a vast number of radiological exams without missing any is challenging.

To address this problem, natural language processing (NLP) could be a help. Indeed, the various applications of NLP in the radiology area have been reported and have shown promising potential [7–14]. Regarding the detection of bone metastasis from radiology reports, rule-based NLP [15, 16], machine learning–based NLP [16], convolutional neural network-based NLP [17], long short-time memory-based NLP [15], and Bidirectional Encoder Representations from Transformers (BERTs)-based fine-tuned large language model (LLM) [16] have been developed. Specifically, BERT, a state-of-the-art approach, exhibiting overwhelming performance across various domains, has shown outperformed performance [16].

However, no report currently applies fine-tuned LLM for detecting bone metastasis based on conditions (progressive or stable/decreased) from unstructured radiology reports. This information is important for stratifying the risk of SREs for clinical physicians and developing a more effective alerting system. Moreover, such a model’s capability to efficiently extract information about patients at risk of SREs from vast data in a short timeframe would enable radiologists engaging SREs to effortlessly and comprehensively identify potential patients in the hospital.

This study aimed to investigate the performance of fine-tuned LLM in extracting patients with progressive bone metastasis in CT from unstructured radiological reports. The comparison study between the model and manual annotation by a radiologist was conducted, assuming practical use where extracting eligible patients potentially needing multidisciplinary approach from a vast amount of radiology reports.

## Materials and Methods

Our Institutional Review Board, which waived the requirement for obtaining written informed consent from patients due to the retrospective study design, approved this retrospective study.

### Datasets

**Fig. 1:**
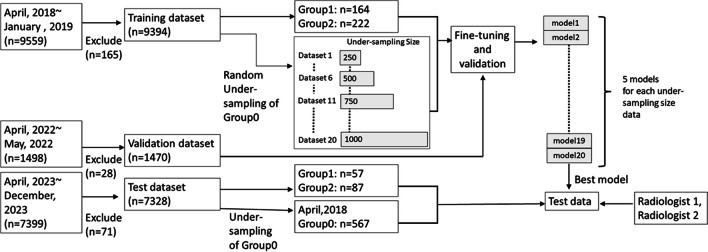
Schematic for data extraction, training, and performance evaluation

The training, validation, and test datasets included radiology reports for CT examinations with various anatomical coverage, including both contrast-enhanced and unenhanced scans. The reports were collected from April 2018 to January 2019, from April to May 2022, and from April 2023 to December 2023, respectively. Reports containing the keyword “metastasis” in either the clinical indication section or imaging diagnosis section were extracted and saved in CSV format. Of the 9559/1498/7399 radiology reports extracted, radiologist A with imaging experience of 4 years reviewed and excluded 165/28/71 due to inadequate information to classify the status, leaving 9394/1470/7328 for training/validation/test datasets, respectively (Fig. 1). All radiological reports were written in Japanese by radiologists with imaging experience of ≥ 5 years.

### Reference Standard

The clinical indication and imaging diagnosis sections of the radiological report were reviewed, and the report was classified into three groups: groups 0 (patients without bone metastasis), 1 (patients with newly identified bone metastasis or an increase in the size of existing bone metastases), and 2 (patients with stable bone metastasis or a decrease in the size or number of existing bone metastases). Radiologist A performed these evaluations for the training, validation, and test. For the test dataset, radiologist B (with imaging experience of 13 years), double-checked the labeling data. Any disagreements were resolved by consensus reading.

### Fine-Tuning of the Pretrained LLM

**Fig. 2:**
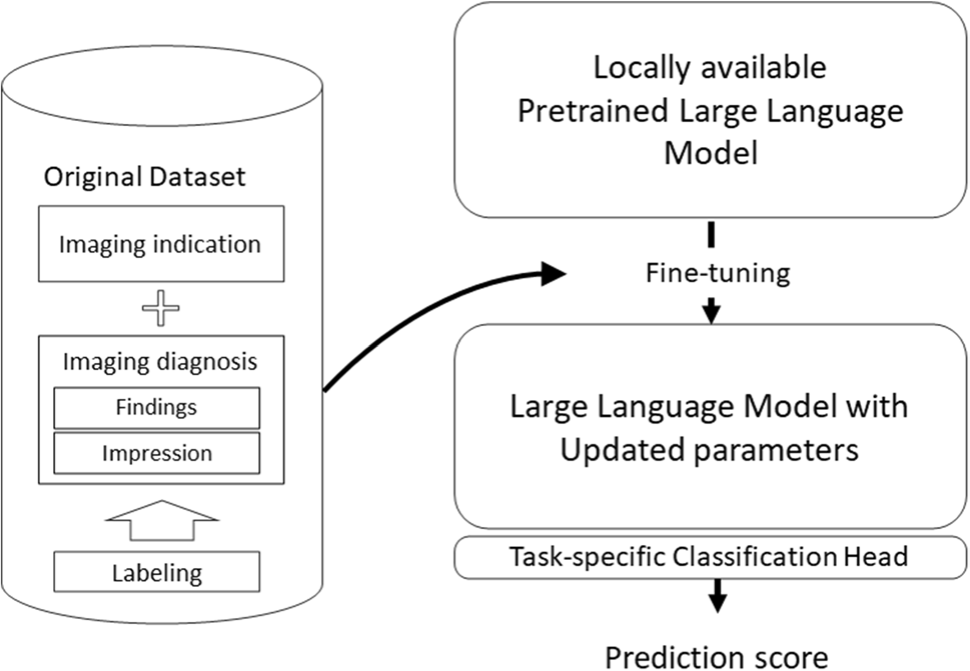
Overview of the fine-tuning of a large language model for report classification

Programming language of Python version 3.10.13 (https://www.python.org/) and Transformers library version 4.35.2 (https://huggingface.co/) on a workstation equipped with a central processing unit of Core™ i9-10980XE, a graphic processing unit of GeForce RTX™ 3060 (NVIDIA), and a random access memory of 64 GB were used to perform fine-tuning of the pretrained BERT Japanese model (https://huggingface.co/cl-tohoku/bert-base-japanese). The model, which consisted of 12 layers, 768 dimensions of hidden states, and 12 attention heads, containing approximately 110 million parameters, was pretrained with Japanese Wikipedia as of September 1, 2019. AutoModelForSequenceClassfication class method in the Transformers library was used to set the model to categorize passages, which consisted of the clinical indication and imaging diagnosis section, into three groups based on the logits for each group (Fig. 2). We conducted training for 20 epochs, specifically sessions 1 through 5, to determine the number of epochs. We randomly under-sampled group 0 into 750 patients out of a total of 9008, considering the negative impact of class imbalance on performance. Our experience using the current dataset indicated the favorability of under-sampling sizes between 500 and 1000; thus, a size of 750 was tentatively selected for this stage. We selected the number of training epochs where the performance on the validation set reached a point of saturation. Other hyperparameters were set at default values of the Transformers library (https://huggingface.co/docs/transformers/main_classes/trainer). An under-sampling technique was used for group 0 by including randomly selected data due to significant class imbalance that may lower sensitivity in groups 1 and 2. Fine-tuning and validation processes were repeated by changing the number of under-sampling sizes (sessions 1, 2, 3, 4, and 5 with under-sampling sizes of 250, 500, 750, and 1000, respectively) to evaluate the effect of under-sampling size of group 0 on the performance. We also conducted the fine-tuning with training data before under-sampling in the same way for comparison. Fine-tuning and validation were conducted in each session. The performance of the non-fine-tuned BERT in the validation dataset was also assessed. The required time for the training, accuracy, sensitivity, and specificity in the validation dataset were recorded. The median of the required time and accuracy in each session were then calculated. The code used for fine-tuning is available upon a reasonable request.

### Test Phase of the Fine-Tuned LLM

The model with the highest accuracy in the validation dataset was further evaluated in the independent test dataset. Group 0 was under-sampled to 567 cases conducted in April 2023, so that the ratio for the number of patients in training/validation/test datasets became 7/1/2. This approach proportionately adjusted the size of the test dataset while mitigating data imbalance to ensure an adequate assessment of minority class. Two other radiologists (readers 1 and 2 with imaging experience of 6 and 1 years, respectively) were involved in manually classifying the reports in the test dataset into the three groups. The classified group data and the time required to complete all the tasks were recorded.

### Statistical Analyses

R version 4.3.2 (https://www.r-project.org/) was used for statistical analyses. For the comparison of continuous variables, an analysis of variance was performed, while the chi-squared test was used for nominal variables. Effect sizes were calculated using eta squared (*η*^2^) for analysis of variance and Cramér’s *V* for the chi-squared test. Inter-rater agreement of the labeling of the test dataset was analyzed using Cohen’s kappa statistics. Kappa values of 0.4–0.6 were interpreted as “moderate,” > 0.6–0.8 as “substantial,” and > 0.8–1 as “almost perfect” [18]. The sensitivity and specificity for each group and accuracy in the test dataset were compared between fine-tuned LLM vs. readers by the McNemar test. The diagnostic performance of the fine-tuned LLM in differentiating group 1 from other groups was evaluated by calculating the area under the receiver operating characteristic curve (AUC) based on probability for this group calculated from logit data. A *p-*value of < 0.050 indicated a statistically significant difference.

## Results

### Datasets

Table 1 shows the distribution of each category in the training, validation, and test datasets. The numbers of patients in groups 0/1/2 were 9008/164/222, 1378/21/71, and 7184/57/87 for the training, validation, and test datasets, respectively. Inter-rater agreement of test datasets revealed “almost perfect” with Cohen’s *κ* = 0.979.

**Table 1:** Distribution of each category across training, validation, and test datasets

	Training	Validation	Test	Comparison
Age (mean ± standard deviation) (years)	66.9 ± 12.6	67.6 ± 12.5	67.9 ± 12.9	< 0.001*
Sex (male/female)	5526/3868	831/639	4255/3073	0.21
Number of reports	9394	1470	7328	
Number of patients in each group				< 0.001*
0: no bone metastasis	9008	1378	7184	
1: progressive bone metastasis	164	21	57	
2: stable or improved bone metastasis	222	71	87	

Comparisons were performed with analysis of variance and chi-squared test for the continuous and nominal variables, respectively. *P*-values are provided in the “Comparison” column P-values. Effect sizes were calculated using eta squared (*η*^2^) for analysis of variance and Cramér’s *V* for the chi-squared test

^*^Note: though there is a statistically significant difference in age and label categories across the groups (*p* < 0.05), the effect size (*η*^2^ = 0.0023 and Cramér’s *V* = 0.0567) is very small, indicating that the practical significance is negligible

### Association Between the Number of Epochs in the Training Dataset and Performance in the Validation Dataset

**Fig. 3:**
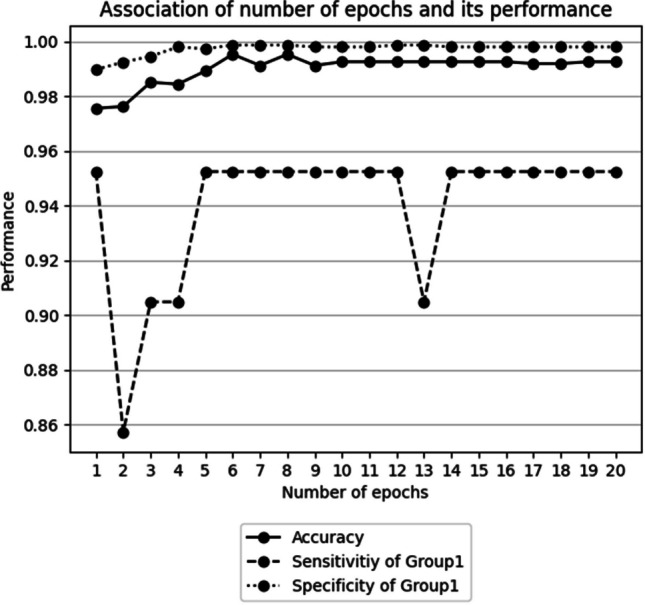
Association between the number of epochs vs. accuracy, sensitivity, and specificity of group

Accuracy and the sensitivity of progressive bone metastasis revealed an increasing trend up to 6 epochs, and they reached an almost plateau over 10 epochs (Fig. 3). Hence, the number of training epochs was set to 10 for subsequent experiments.

### Effect of Under-Sampling on the Sensitivity for Each Group in the Validation Dataset

**Fig. 4:**
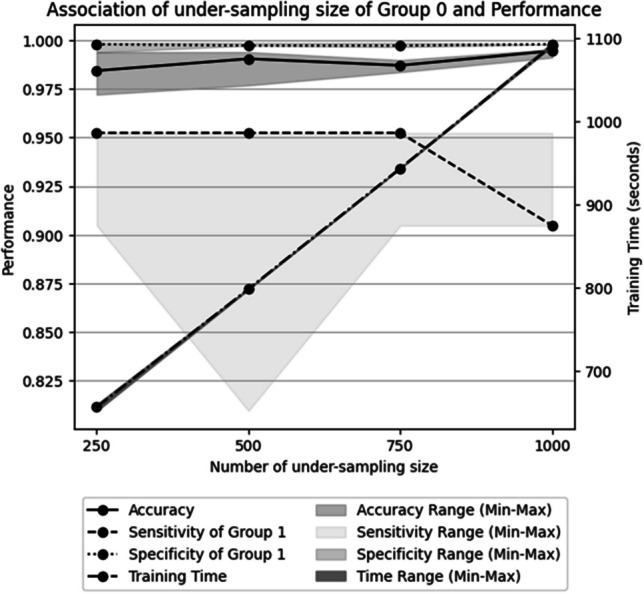
Association between the under-sampling size of group 0 in the training dataset vs. accuracy, sensitivity, and specificity of group 1 in the validation dataset and the required time for the training. The median metrics and range (max and min) among five models for each under-sampling size are provided by dots and gray lines, respectively

The accuracy and sensitivity of group 1 of the non-fine-tuned model were 0.937 and 0.000, respectively. For the fine-tuned model with no under-sampling of the training data, the median accuracy and median sensitivity for group 1 were also 0.937 and 0.000, respectively. An under-sampling technique was used for group 0 by including randomly selected data due to class imbalance that may lower sensitivity in groups 1 and 2. The median accuracy of the model was 0.984/0.990/0.987/0.994, and the sensitivity of group 1 was 0.952/0.952/0.952/0.905 (under-sampling size = 250/500/750/1000, respectively, Fig. 4).

Each model of sessions 1–5 with under-sampling sizes of 250/500/750/1000 was reviewed, and the model with the highest accuracy was selected for further performance evaluation in the test dataset.

Among 20 models, the one with an under-sampling size of 500 demonstrated the highest performance, with sensitivities for each group at 0.996, 0.952, and 0.972, and class accuracies at 0.996, 0.998, and 0.994, respectively.

### Performance of the Fine-Tuned LLM and Radiologists in the Test Dataset

Table 2 shows a confusion matrix for the reference standard vs. prediction data by the best-fine-tuned LLM and radiologists. Table 3 presents sensitivity, accuracy, and specificity data. The accuracy of the fine-tuned LLM (0.979) was slightly lower than that of readers 1 (0.996) and 2 (0.992). The sensitivity of groups 1 and 2 of the fine-tuned LLM (0.947, 0.943) was comparable to that of readers 1 (1, 0.966) and 2 (0.982, 0.954). The specificity of group 0 of the fine-tuned LLM (0.993) was superior to that of reader 1 (0.986) and comparable to reader 2 (0.993).

**Table 2:** Confusion matrix for reference standard *vs.* prediction data

	Reference standard		
	Group 0 (*n* = 567)	Group 1 (*n* = 57)	Group 2 (*n* = 87)
Large language model			
Group 0 (*n* = 561)	560	0	1
Group 1 (*n* = 60)	2	54	4
Group 2 (*n* = 90)	5	3	82
Reader 1			
Group 0 (*n* = 569)	567	0	2
Group 1 (*n* = 58)	0	57	1
Group 2 (*n* = 84)	0	0	84
Reader 2			
Group 0 (*n* = 568)	567	0	1
Group 1 (*n* = 59)	0	56	3
Group 2 (*n* = 84)	0	1	83

**Table 3:** Accuracy, sensitivity, specificity, and time required data in the test dataset

	Fine-tuned LLM	Reader 1		Reader 2	
		Score	Comparison	Score	Comparison
Accuracy	0.979 (696/711)	0.996 (708/711)	*p* < 0.05	0.993 (706/711)	*p* < 0.05
Sensitivity for each group					
Group 0	0.988 (560/567)	1.000 (567/567)	*p* < 0.05	1.000 (567/567)	*p* < 0.05
Group 1	0.947 (54/57)	1.000 (57/57)	0.248	0.982 (56/57)	0.480
Group 2	0.943 (82/87)	0.966 (84/87)	0.683	0.954 (83/87)	1.000
Specificity for each group					
Group 0	0.993 (143/144)	0.986 (142/144)	0.182	0.993 (143/144)	0.547
Group 1	0.991 (648/654)	0.998 (653/654)	*p* < 0.05	0.995 (651/654)	0.080
Group 2	0.987 (616/624)	1.000 (624/624)	*p* < 0.05	0.998 (623/624)	*p* < 0.05
Precision for each group					
Group 0	0.988 (560/567)	0.997 (567/569)		0.998 (567/568)	
Group 1	0.915 (54/59)	0.983 (57/58)		0.949 (56/59)	
Group 2	0.943 (82/87)	1.0 (84/84)		0.988 (83/84)	
Time required (s)	105	2312		3094	

Comparisons between fine-tuned LLM *vs.* readers were performed with the McNemar test

*LLM* large language model

**Fig. 5:**
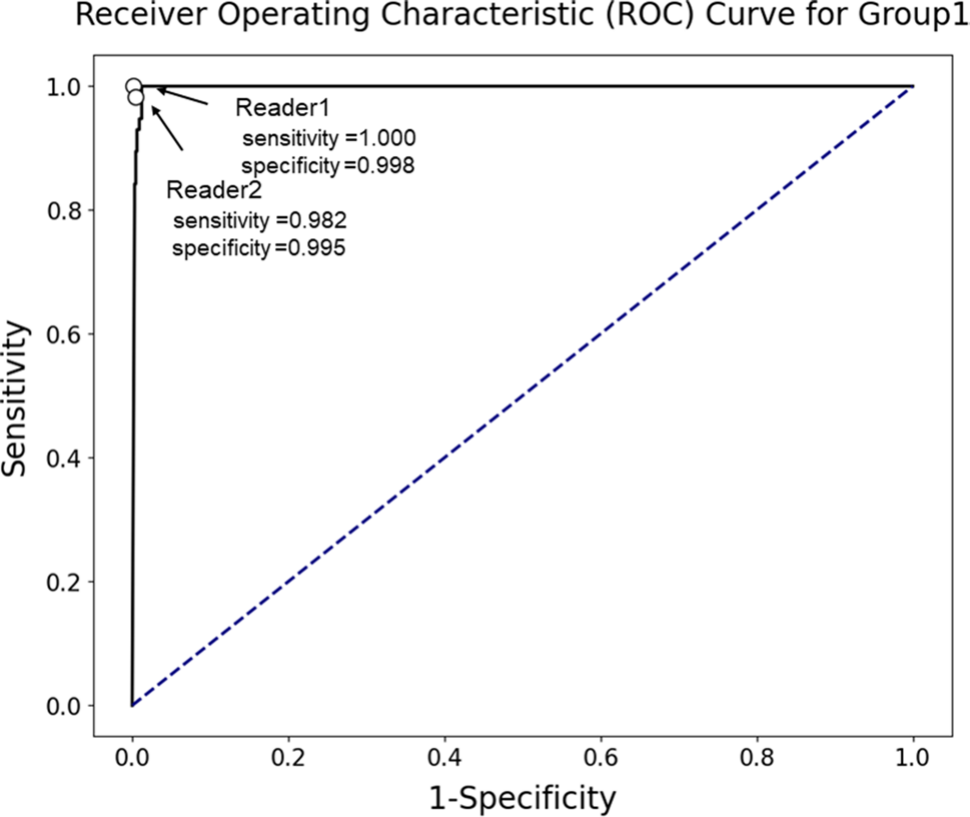
Receiver operating characteristic curve for fine-tuned large language model in discriminating group 1 (progressive bone metastasis) from other groups in the test dataset. The area under the receiver operating characteristic curve was 0.997. Radiologists’ performance was plotted

**Fig. 6:**
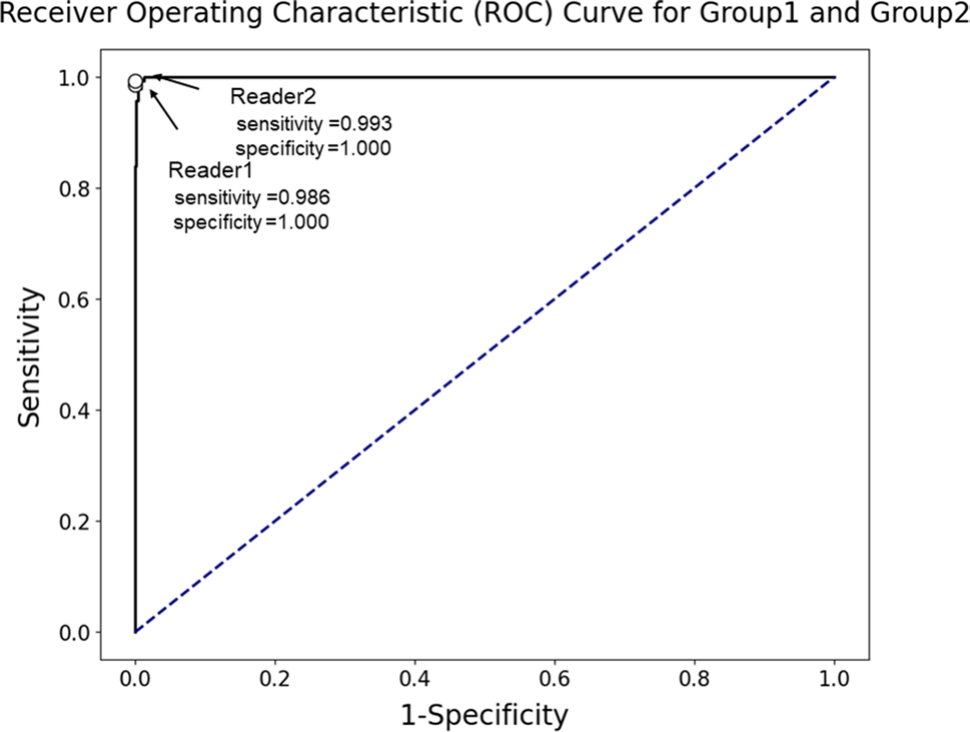
Receiver operating characteristic curve for fine-tuned large language model in discriminating groups 1 and 2 from group 0, encompassing all bone metastases irrespective of their status (progressive, stable, or decreased), in the test dataset. The area under the receiver operating characteristic curve was 1.000. Radiologists’ performance was plotted

The diagnostic performance for discriminating group 1 from other groups using output probability for this group, evaluated with AUC, was 0.997 (95% confidence interval, 0.988–1.000) (Fig. 5). Additionally, diagnostic performance assessment for discriminating groups 1 and 2 from group 0, encompassing all bone metastases irrespective of their status (progressive, stable, or decreased), revealed an AUC of 1.000 (95% confidence interval, 0.997–1.000) (Fig. 6).

The time required for LLM classification of the test dataset (*n* = 711) was 22.0–29.5 times shorter than that of readers 1 and 2 (105 s vs. 2312 and 3094 s, respectively).

## Discussion

This study assessed the feasibility of a fine-tuned LLM for detecting progressive bone metastasis from unstructured radiology reports. Our best-fine-tuned model demonstrated excellent performance in the context of progressive bone metastasis. Moreover, the inference time for our fine-tuned LLM demonstrated a remarkable reduction than the manual annotation.

Several natural language models for detecting bone metastasis from radiology reports have been developed, including rule-based NLP [15, 16], machine learning–based NLP [16], convolutional neural network-based NLP [17], long short-term memory-based NLP model [15], and a BERT-based LLM [16]. In this study, we selected the fine-tuned BERT-based LLM for several reasons. First, BERT represents a state-of-the-art approach, exhibiting overwhelming performance across various domains. Do et al. developed BERT-based NLP, rule-based NLP, and machine learning–based NLP models, with the BERT-based NLP model demonstrating the highest performance [16]. Second, a fine-tuned LLM is easily obtained by fine-tuning a publicly available pretrained LLM with limited graphic processing unit resources in a short training time (799 s for our best model), without requiring extensive rule-making or complicated model programming. Finally, a locally deployed pretrained LLM was fine-tuned locally, ensuring privacy and security, which is a significant advantage in the medical domain [19]. This contrasts with models like ChatGPT or GPT-4, which require data uploading to third-party servers for fine-tuning. Our model demonstrates higher accuracy and sensitivity (0.979 and 0.943–0.988) compared to the previously reported BERT model with an accuracy of 0.96, precision of 0.88, and sensitivity of 0.75 [16]. However, this result was not directly comparable due to the differences in the included text data (only the diagnosis section or both the diagnosis section and clinical indication section), variations in language within the reports (English or Japanese), alterations in report structure (unstructured or structured), patient demographics.

The presented model demonstrated acceptable performance, not only in detecting bone metastasis but also in identifying cases of progressive bone metastasis with significantly shorter inference times compared to manual annotation. This indicates the model’s potential for practical clinical applications, particularly in developing alerting systems to notify clinical physicians about patients at a heightened risk of SREs. Furthermore, this model holds promise not only for alert system for referring physician but also for supporting a multidisciplinary approach to patients facing an elevated risk of SREs. Radiologists effortlessly and comprehensively identify and review potential patients under their care in the hospital, facilitating smooth integration into multidisciplinary discussions and prompt interventions, considering the model’s capability to efficiently extract information about patients with bone metastasis from the large data in a short timeframe. Similarly, it can be used in the research setting to extract eligible patients in future investigations, particularly in large-scale studies, such as those related to deep learning model development and machine learning models with radiomics features.

Class imbalance is known to cause uneven sensitivity across different groups [20, 21]. Common methods to address this issue include under-sampling and oversampling (such as synonym replacement [22], back-translation [23], and translation augmentation [24], as well as ensembling methods [20, 25]) techniques. Oversampling can lead to overfitting, where the model becomes overly specialized to the minority class and performs poorly on unseen data, especially in cases of highly skewed data distributions (e.g., our data with 9008/164/222 for group 0/1/2). Given these concerns, we opted for under-sampling techniques. However, under-sampling may result in the loss of valuable information from the majority class, particularly if the sampling process is biased. To address this, we developed five models using under-sampling for group 0 data, taking into account the variability introduced by the random under-sampling process, and selected the best model. The application of the under-sampling technique improved the sensitivity for group 0 without compromising the sensitivity for group 1 or group 2 in the validation dataset.

As for data extraction, we used the keyword “metastasis” instead of “bone metastasis” and included all cases regardless of their association with cancer, provided there was adequate information to classify the status of bone metastasis. The reason for using “metastasis” rather than “bone metastasis” is that bone metastasis is sometimes described using terms like “vertebral metastasis,” which we interpreted as bone metastasis in context. By using the broader keyword “metastasis,” we were able to capture a wider range of relevant cases, which we believe was crucial for developing a robust model. This approach allowed us to include diverse terms related to bone metastasis, enhancing the model’s applicability and accuracy.

This study has some limitations. First, the model was developed and evaluated exclusively with our institutional dataset; thus, its performance on external datasets remains unknown. However, notably, our study primarily aimed not to present a model directly applicable to external institutions. Instead, we aim to demonstrate that high performance customized LLMs can be easily developed within each institution. This study’s dataset included a broad spectrum of cancers, considering our hospital’s role as a core center of medical science and care. Additionally, the achievement of high performance despite such diversity adds a positive aspect to the feasibility of this approach. Second, our model considered only size change for the criteria of impeding bone metastasis and did not consider other possible important risk factors for SREs such as site, type (lytic, blastic, or mixed) of the lesion, cortical involvement, and systemic treatment causing osteoporosis, such as hormone therapy, chemotherapy, and steroids [2, 26, 27]. The future work should consider the factor to further stratify the risk of SREs. Third, we used the BERT Japanese model as our LLM. Recognizing that our results may not necessarily extend to other languages is crucial, considering the potential variability in model performance across different languages. Finally, the sensitivity of progressive bone metastasis was 0.947, indicating that not all cases were successfully identified. Caution is warranted when considering model application in practical clinical situations.

## Conclusion

The fine-tuned large language model effectively extracts not only bone metastasis but also progressive bone metastasis from the picture archiving and communication system. Its performance is comparable or slightly lower when compared to manual annotation, yet the performance is acceptable and the inference time is significantly faster. The fine-tuned LLM exhibits promising potential for application in clinical settings as an alerting system, contributing to serious skeletal-related event prevention.

## Data Availability

The datasets generated and/or analyzed during the current study are not publicly available due to patients' confidentiality.

## References

[1] Ibrahim T, Flamini E, Fabbri L, Serra P, Mercatali L, Ricci R, Sacanna E, Falasconi M.C, Casadei R, Galassi R, Giannini M, Bazzocchi O, Calzolari F, Nunziatini R, Gaudio M, Maltoni M, Amadori D. Multidisciplinary approach to the treatment of bone metastases: Osteo-Oncology Center, a new organizational model. Tumori. 95:291–297, 2009 10.1177/030089160909500304 19688966

[2] Kimura T. Multidisciplinary Approach for Bone Metastasis: A Review. Cancers (Basel). 10:1–10, 2018 10.3390/cancers10060156 PMC6025143 29795015

[3] Sloan CE, Chadalavada SC, Cook TS, Langlotz CP, Schnall MD, Zafar HM. Assessment of follow-up completeness and notification preferences for imaging findings of possible cancer: what happens after radiologists submit their reports? Acad Radiol. 21:1579–1586, 2014. 10.1016/j.acra.2014.07.006 PMC4825815 25179562

[4] Callen JL, Westbrook JI, Georgiou A, Li J. Failure to follow-up test results for ambulatory patients: A systematic review. J Gen Intern Med. 27:1334–1348, 2012. 10.1007/s11606-011-1949-5 PMC3445672 22183961

[5] Solberg A, Bremnes RM. Metastatic spinal cord compression: diagnostic delay, treatment, and outcome. Anticancer Res. 19:677–684, 1999. 10216476

[6] Loven D, Gørnish M, Fenig GE, Sulkes A, Rappaport Z, Klir I, Rotenberg Z, Gadoth N. [Malignant epidural cord compression]. Harefuah. 131:457–462, 1996. 9043151

[7] Nakamura Y, Hanaoka S, Nomura Y, Nakao T, Miki S, Watadani T, Yoshikawa T, Hayashi N, Abe O. Automatic detection of actionable radiology reports using bidirectional encoder representations from transformers. BMC Med Inform Decis Mak. 21:262,2021 10.1186/s12911-021-01623-6 PMC8436473 34511100

[8] Adams L C, Truhn D, Busch F, Kader A, Niehues S M, Makowski M R, Bressem K K. Leveraging GPT-4 for Post Hoc Transformation of Free-text Radiology Reports into Structured Reporting: A Multilingual Feasibility Study. Radiology. 10.1148/radiol.230725, Apr 4, 2023. 10.1148/radiol.230725 37014240

[9] Mukherjee P, Hou B, Lanfredi RB, Summers RM. Feasibility of Using the Privacy-preserving Large Language Model Vicuna for Labeling Radiology Reports. Radiology. 10.1148/radiol.231147, October 10,2023. 10.1148/radiol.231147 PMC10623189 37815442

[10] Yasaka K, Kanzawa J, Kanemaru N, Koshino S, Abe O. Fine Tuned Large Language Model for Extracting Patients on Pretreatment for Lung Cancer from a Picture Archiving and Communication System Based on Radiological Reports. J Imaging Informatics Med. 10.1007/s10278-024-01186-8, July 2, 2024. 10.1007/s10278-024-01186-8 PMC11811339 38955964

[11] Eghbali N, Siegal D, Klochko C, Ghassemi MM. Automation of Protocoling Advanced MSK Examinations Using Natural Language Processing Techniques. AMIA Jt Summits Transl Sci. 2023:118–127, 2023. PMC10283088 37350898

[12] Wong KA, Hatef A, Ryu JL, Nguyen X V., Makary MS, Prevedello LM. An Artificial Intelligence Tool for Clinical Decision Support and Protocol Selection for Brain MRI. Am J Neuroradiol. 44:11–16, 2023 10.3174/ajnr.A7736 PMC9835923 36521960

[13] Talebi S, Tong E, Li A, Yamin G, Zaharchuk G, Mofrad MRK. Exploring the performance and explainability of fine-tuned BERT models for neuroradiology protocol assignment. BMC Med Inform Decis Mak. 24:1–12, 2024 10.1186/s12911-024-02444-z PMC10848624 38326769

[14] Kanzawa J, Yasaka K, Fujita N, Fujiwara S, Abe O. Automated classification of brain MRI reports using fine-tuned large language models. Neuroradiology. 10.1007/s00234-024-03427-7. July 12, 2024. 10.1007/s00234-024-03427-7 PMC11611921 38995393

[15] Doi K, Takegawa H, Yui M, Anetai Y, Koike Y, Nakamura S, Tanigawa N, Koziumi M, Nishio T. Deep learning-based detection of patients with bone metastasis from Japanese radiology reports. Jpn J Radiol. 41:900–908, 2023 10.1007/s11604-023-01413-2 36988827

[16] Do RKG, Lupton K, Causa Andrieu PI, Luthra A, Taya M, Batch K, Nguyen H, Rahurkar P, Gazit L, Nicholas K, Fong CJ, Gangai N, Schultz N, Zulkernine F, Sevilimedu V, Juluru K, Simpson A, Hricak H. Patterns of Metastatic Disease in Patients with Cancer Derived from Natural Language Processing of Structured CT Radiology Reports over a 10-year Period. Radiology. 301:115–122, 2021. 10.1148/radiol.2021210043 PMC8474969 34342503

[17] Kehl KL, Elmarakeby H, Nishino M, Van Allen EM, Lepisto EM, Hassett MJ, Johnson BE, Schrag D. Assessment of Deep Natural Language Processing in Ascertaining Oncologic Outcomes From Radiology Reports. JAMA Oncol. 5:1421–1429, 2019. 10.1001/jamaoncol.2019.1800 PMC6659158 31343664

[18] Landis JR, Koch GG. Agreement of categorical data. Biometrics. 33:159–174, 1977 843571

[19] Cai W. Feasibility and Prospect of Privacy-preserving Large Language Models in Radiology. Radiology. 10.1148/radiol.232335, October10, 2023. 10.1148/radiol.232335 PMC10623203 37815443

[20] Xavier BA, Chen PH. Natural Language Processing for Imaging Protocol Assignment: Machine Learning for Multiclass Classification of Abdominal CT Protocols Using Indication Text Data. J Digit Imaging. 35:1120–1130, 2022. 10.1007/s10278-022-00633-8 PMC9582109 35654878

[21] Yasaka K, Akai H, Abe O, Kiryu S. Deep learning with CNN showed high diagnostic performance in differentiation of liver masses at dynamic CT. Radiology. 286:887-896, 2018. 10.1148/radiol.2017170706 29059036

[22] Wei J, Zou K. EDA: Easy data augmentation techniques for boosting performance on text classification tasks. EMNLP-IJCNLP 2019, 10.18653/v1/d19-1670, 2019.

[23] Sennrich R, Haddow B, Birch A. Improving neural machine translation models with monolingual data. In Proceedings of the 54th Annual Meeting of the Association for Computational Linguistics, 10.18653/v1/p16-1009, August 2018.

[24] Fadaee M, Bisazza A, Monz C. Data augmentation for low-Resource neural machine translation. In Proceedings of the 55th Annual Meeting of the Association for Computational Linguistics, 10.18653/v1/P17-2090, July 2017.

[25] Olthof AW, van Ooijen PMA, Cornelissen LJ. Deep Learning-Based Natural Language Processing in Radiology: The Impact of Report Complexity, Disease Prevalence, Dataset Size, and Algorithm Type on Model Performance. J Med Syst. 45(91), 2021. 10.1007/s10916-021-01761-4 PMC8416876 34480231

[26] Mirels H. Metastatic disease in long bones. A proposed scoring system for diagnosing impending pathologic fractures. Clin Orthop Relat Res. 249:256–264. 1989. 2684463

[27] Van der Linden YM, Dijkstra PDS, Kroon HM, Lok JJ, Noordijk EM, Leer JW, Marijnen CA. Comparative analysis of risk factors for pathological fracture with femoral metastases. J Bone Joint Surg Br. 86:566–573, 2004. 15174555

